# Arsenic Induces Functional Re-Expression of Estrogen Receptor α by Demethylation of DNA in Estrogen Receptor-Negative Human Breast Cancer

**DOI:** 10.1371/journal.pone.0035957

**Published:** 2012-04-27

**Authors:** Juan Du, Nannan Zhou, Hongxia Liu, Fei Jiang, Yubang Wang, Chunyan Hu, Hong Qi, Caiyun Zhong, Xinru Wang, Zhong Li

**Affiliations:** 1 State Key Laboratory of Reproductive Medicine, Institute of Toxicology, Nanjing Medical University, Nanjing, China; 2 Key Laboratory of Modern Toxicology (Ministry of Education), School of Public Health, Nanjing Medical University, Nanjing, China; 3 Department of Nutrition and Food Hygiene, School of Public Health, Nanjing Medical University, Nanjing, China; 4 The Safety Assessment and Research Center for Drugs, Nanjing, Jiangsu Province, China; King Faisal Specialist Hospital & Research center, Saudi Arabia

## Abstract

Estrogen receptor α (ERα) is a marker predictive for response of breast cancers to endocrine therapy. About 30% of breast cancers, however, are hormone- independent because of lack of ERα expression. New strategies are needed for re-expression of ERα and sensitization of ER-negative breast cancer cells to selective ER modulators. The present report shows that arsenic trioxide induces reactivated ERα, providing a target for therapy with ER antagonists. Exposure of ER-negative breast cancer cells to arsenic trioxide leads to re-expression of ERα mRNA and functional ERα protein in *in vitro* and *in vivo*. Luciferase reporter gene assays and 3-(4,5-dimethylthiazol-2-yl)- 5-(3-carboxymethoxyphenyl)- 2-(4-sulfophenyl)- 2H-tetrazolium (MTS) assays show that, upon exposure to arsenic trioxide, formerly unresponsive, ER-negative MDA-MB-231 breast cancer cells become responsive to ER antagonists, 4-hydroxytamoxifen and ICI 182,780. Furthermore, methylation- specific PCR and bisulfite-sequencing PCR assays show that arsenic trioxide induces partial demethylation of the ERα promoter. A methyl donor, S-adenosylmethionine (SAM), reduces the degree of arsenic trioxide-induced re-expression of ERα and demethylation. Moreover, Western blot and ChIP assays show that arsenic trioxide represses expression of DNMT1 and DNMT3a along with partial dissociation of DNMT1 from the ERα promoter. Thus, arsenic trioxide exhibits a previously undefined function which induces re-expression ERα in ER-negative breast cancer cells through demethylation of the ERα promoter. These findings could provide important information regarding the application of therapeutic agents targeting epigenetic changes in breast cancers and potential implication of arsenic trioxide as a new drug for the treatment of ER–negative human breast cancer.

## Introduction

Breast cancer is the most common tumor in women, and it is the cause of considerable morbidity and mortality. The tumor expression of estrogen receptors (ERs) is a marker for prognosis and is predictive of response to endocrine therapy [1]. Approximately two-thirds of breast cancers express ER gene and synthesize ER protein. These tumors tend to be more differentiated and are often responsive to hormonal therapies. One-third of breast cancers, however, lack ER. Loss of ER expression is generally associated with poor histological differentiation, high growth fractions, and inferior clinical outcomes [2]. These cancers, which are apparently estrogen-independent, rarely respond to hormonal therapies. In addition, some breast cancers that are initially ER-positive lose ER expression during tumor progression [3].

To date, the molecular mechanisms underlying the loss of ERs in breast cancer are poorly understood. Deletions, insertions, rearrangements, or polymorphisms of ER genes are infrequent and are not generally associated with loss of ER [4]. It has been shown that breast cancer, similar to other types of cancer, is also a disease that is driven by epigenetic alterations, which do not affect the primary DNA sequence [5], [6]. Epigenetic alterations include DNA methylation, histone modifications and so on. In human breast carcinomas, an abnormal methylation pattern could account for transcriptional inactivation of the ER gene and subsequent hormone resistance. Loss of ER is premodinantly a result of hypermethylation of CpG islands, cytosine–guanine-rich areas that are located in the 5′ regulatory regions of the ER gene [2], [7], [8]. Theoretically, if the elimination of ERα expression is a consequence of hypermethylation of these islands, this effect could be reversed by a DNA demethylating agent. Other epigenetic events, such as deacetylation and methylation of histones, are also involved in the complex mechanisms that regulate promoter transcription. Demethylating agents and histone deacetylase (HDAC) inhibitors, which are candidates for new cancer therapeutics, can produce a synergistic reactivation to restore ERα protein expression in ER–negative breast cancer cells [3].

Arsenic trioxide, which has been used clinically as an anti-tumor agent, induces demethylation and apoptosis [9], while little is known regarding the mechanism of its anti-tumor effect. Intracellular arsenic metabolism and DNA methylation require S-adenosylmethionine (SAM) as a methyl donor, and the consumption of methyl groups in arsenic biotransformation presumably results in a deficiency of methyl donors, reducing DNA methylation [10]. Thus, arsenic trioxide possibly induces demethylation and re-expression of certain genes. To date, however, whether arsenic trioxide can re-express ERα in ER-negative breast cancer cells has not been investigated. In the present study, ER–negative human breast cancer cells were exposed to arsenic trioxide to test the hypothesis that ERα promoter hypermethylation could be reversed, leading to reactivation of the ERα gene. We thought that data from our study could provide important information regarding the potential implication of arsenic trioxide as a new drug for treatment of ER–negative human breast cancer.

## Materials and Methods

### Chemicals, reagents, and supplies

Arsenic trioxide, β-estradiol (E_2_), 4-hydroxytamoxifen (OHT),ICI 182,780 (ICI) and 5-*aza*-2′-deoxycytidine (AZA) were purchased from Sigma Chemical Co. (St. Louis, MO, USA). SAM was from New England BioLabs. TRIzol reagent, L-15 medium, Dulbecco's modified Eagle's medium (DMEM), and trypsin were from Gibco/Invitrogen (Carlsbad, CA, USA). DNeasy Blood & Tissue kits were obtained from QIAGEN (Alameda, CA, USA). CpGenome DNA modification kits and Magna ChIP^TM^ G One-Day Chromatin Immunoprecipitation Kits were purchased from Millipore (Billerica, MA, USA). Fetal bovine serum (FBS) was obtained from PAA Laboratories (Pasching, Austria) and charcoal-stripped FBS (CS-FBS) from Biological Industries (Kibbutz Beit Haemek, Israel).

Rabbit polyclonal antibodies against ERα, β-actin, and GAPDH were obtained from NeoMarkers (Fremont, CA, USA), and the mouse monoclonal antibodies against DNMT1,DNMT3a and DNMT3b was obtained from Abnova (Taipei, Taiwan). Anti-rabbit and anti-mouse peroxidase-conjugated antibodies were obtained from Upstate Biotechnology (Charlottesville, VA, USA). Sheep polyclonal antibodies against MBD2 were obtained from Millipore (Billerica, MA, USA). The pEASY-T3 Cloning Kit was from Beijing TransGen Biotech Co. (Beijing, China). Enhanced chemiluminescence detection reagents were obtained from Amersham Biosciences (Piscataway, NJ, USA). The tetrazolium compound, 3-(4,5-dimethylthiazol-2-yl)-5– (3-carboxymethoxyphenyl)-2-(4-sulfophenyl)-2H-tetrazolium (MTS) and lysis buffer were obtained from Promega Biotech Co. (Beijing, China). The Sofast™ transfection reagent was purchased from Sunma Co. (Xiamen, China). Methyl Primer Express software was purchased from Applied Biosystems (Foster City, CA, USA).

### Plasmids

The luciferase reporter plasmid pERE-TATA-Luc+ and rat ERα expression vector rERa/pCI were provided by Dr. M. Takeyoshi (Chemicals Assessment Center, Chemicals Evaluation and Research Institute, Oita, Japan). The plasmids were constructed as previously described [11]. The plasmid phRL-tk containing the Renilla luciferase gene (used as an internal control for transfection efficiency and cytotoxicity of test chemicals) and the Dual-Luciferase Reporter Assay System Kit were purchased from Promega (Madison, WI, USA).

### Cell culture and treatments

Human breast cancer cells, MDA-MB-231, Hs578T and MCF-7, were obtained from American Type Culture Collection (Rockville, MD, USA), and were maintained according to ATCC's recommendation. MDA-MB-231 cells were maintained in L-15 medium; MCF-7 and Hs578T cells were routinely maintained in DMEM. Both media were supplemented with 100 IU/mL penicillin, 100 mg/mL streptomycin, and 10% FBS. MCF-7 and Hs 578T cells were incubated at 37°C in 5% CO_2_; MDA-MB-231 cells were grown in the absence of CO_2_. Cells were seeded at a density of 2×10^5^ per 100-mm tissue culture dish in phenol red–free DMEM supplemented with 10% CS-FBS (estrogen-free), and the medium was changed every other day for 6 days. After 24 hr, the estrogen-free medium was changed to estrogen-free medium containing 1, 2, or 4 μmol/L of arsenic trioxide or 200 μmol/L of SAM for 6 days. Cells untreated or pretreated with arsenic trioxide/SAM were exposed to E_2_ (100 nmol/L for 24 hr), OHT (10 μmol/L for 24 hr), ICI (2 μmol/L for 24 hr), or to the vehicle in fresh estrogen-free medium for indicated time periods. The media, with or without added compounds, were renewed every second day. For cell proliferation assays, reverse transcription polymerase chain reactions (RT-PCR), and reporter gene analysis, cells were switched to phenol red-free MEM (without E_2_) supplemented with 5% CS-FBS at least 5 days before exposure E_2_, OHT, or ICI. The experiments were repeated twice, and each cell line was tested in triplicate.

### Cell growth assay

MDA-MB-231 cells were plated at a density of 4×10^3^ cells/well into 96-well plates and cultured with phenol red-free MEM supplemented with 5% CS-FBS. After 24 hr, the medium was replaced, and the cells were exposed to arsenic trioxide for 6 days. Cells untreated or pretreated with arsenic trioxide were exposed to E_2_, OHT, ICI, or vehicle in fresh estrogen-free medium for indicated time periods. At appropriate times, MTS was added to each well, and the plates were further incubated for 4 hr at 37°C. The absorbance at 490 nm was measured with a Multiscan MCC 340 microplate reader (Titertek, Huntsville, AL, USA). All measurements were performed in triplicate. Data points represented the means of the values for the four wells. Cellular proliferation was expressed as the mean numbers of cells ± SEM.

### Nude mice xenograft model

Female nude mice (6 weeks old; BALB/cA-nu (nu/nu)) were purchased from Shanghai Laboratory Animal Center (SLAC, Shanghai, China) and maintained in pathogen-free conditions. The use and care of animals in this study is approved by the Institutional Animal Care and Use Committee of Nanjing Medical University (Approval ID 20100234). MDA-MB-231 cells (1×10^7^ cells) were injected into both flanks of each nude mouse, and tumors were allowed to grow for 6 weeks. Ten mice were randomly divided into 2 groups. For arsenic trioxide-treated group, 2 mg/kg.bw arsenic trioxide (The dose was selected based on the commonly used clinical dose of 10 mg/d which was converted to the dose in nude mice) was administered i.p. in 100 μl of sterile saline every other day. For control group, the nude mice were treated with the same volume of PBS. Mice were sacrificed after 4 weeks of treatments. Tumors were removed from the mice, immediately frozen on dry ice and stored in liquid nitrogen. Tumors were also fixed with 10% formalin solution for immunohistochemical staining.

### RNA isolation and RT-PCR

Cells were grown in 6-cm plastic dishes (1×10^6^ cells/dish in 5 mL of estrogen-free medium). At 24 hr after plating, the test compounds were added. Total cellular RNA was extracted by use of the TRIzol reagent (Invitrogen, Carlsbad, CA, USA) and quantified by UV absorption. From each sample, 1 μg was reverse-transcribed by M-MLV first-stand amplification of ERα, pS2 and GREB1 (estrogen-responsive genes), and the constitutively expressed housekeeping gene, GAPDH. Primers for ERα were 5′-CCACCAACCAGTGCACCATT-3′ (forward) and 5′-GTCTTTCCG TATCCCACCTTTC-3′ (reverse). Primers for pS2 and GREB1 and the PCR conditions have been described previously [12]. The PCR products were resolved by 2% agarose gel electrophoresis and detected by UV transillumination after staining with ethidium bromide.

### Western blot analyses

The cells were washed twice with ice-cold PBS, scraped into 0.2 mL of buffer [20 mM HEPES (pH 6.8), 5 mM EDTA, 10 mM EGTA, 5 mM NaF, 0.1 μg/mL okadaic acid, 1 mM dithiothreitol, 0.4 M KCl, 0.4% Triton X-100, 10% glycerol, 5 μg/mL leupeptin, 50 μg/mL of phenylmethanesulphonylfluoride, 1 mM benzamidine, 5 mg/mL aprotinin and 1 mM sodium orthovanadate], and incubated on ice for 30 min, followed by centrifugation at 12,000 rpm for 15 min. The supernatant was stored at −70°C. Protein concentrations were measured with the BCA Protein Assay (Pierce, Rockford, IL, USA). Afterwards, proteins were diluted to equal concentrations, boiled for 5 min, and separated by 7%–12% sodium dodecyl sulfate–polyacrylamide gel electrophoresis. Proteins were transferred to nitrocellulose membranes, which were probed with ERα and DNMT1 antibodies overnight at 4°C. Membranes were incubated with horseradish peroxidase-conjugated secondary antibodies for 1 hr at room temperature to enhance chemiluminescence (Amersham Biosciences, Piscataway, NJ, USA) before exposure to film. GAPDH or β-actin was used to normalize for protein loading. All experiments were performed at least twice; similar results were obtained.

### Transfection and luciferase reporter gene assays

MDA-MB-231 cells were placed in 24-well microplates at a density of 5.0×10^3^ cells/well in the phenol-red-free MEM containing 5% CS-FBS. Following 24 hr of incubation, the cells were exposed to arsenic trioxide for 6 days, then transfected with 0.5 μg of pERE-TATA-Luc+, 0.2 μg of rERa/pCI, and 0.1 μg of phRL-tk, with 5 μg of Sofast™ transfection reagent per well. After incubation for 12 hr, the transfection medium was replaced. After being exposed to the test chemicals for 24 hr, the cells were harvested. Following three rinses with PBS (pH 7.4), the cells were lysed with 1× passive lysis buffer. The cell lysates were analyzed immediately with a 96-well plate luminometer. The amounts of luciferase and Renilla luciferase were measured with the Dual-Luciferase Reporter Assay System Kit following the manufacturer's instructions. The value of luciferase activity for each lysate was normalized to the Renilla luciferase activity. The relative transcriptional activity was converted into fold induction above the vehicle control value (n-fold).

### Bisulfite sequencing PCR (BSP)

Genomic DNA was isolated by use of a DNeasy Blood & Tissue kit. Isolated DNA was subjected to modification by use of a CpGenome DNA modification kit according to the manufacturer's recommendations and amplified via PCR with primers for the ERα promoter region. From the bisulfite-modified DNA, the ERα promoter was amplified by PCR under conditions described previously [12]. ERS primers were 5′-TGTTTGGAGTGATGTTTAAGTT-3′ and 5′- CAATAAAACCATCCCAAATACT-3′. PCR products were gel purified and cloned using the pEASY-T3 Cloning Kit (Beijing TransGen Biotech Co., Ltd.). Ten random clones were sequenced by the Invitrogen Company.

### Methylation-specific PCR (MSP) analysis

The ER gene promoter was amplified from bisulfite-modified DNA by two rounds of PCR using nested primers specific to the bisulfite-modified sequence of the CpG island of the ER gene. The first-round PCR involved BSP primers; the second-round primers specifically recognized the unmethylated or methylated promoter sequences after bisulfite conversion. ER unmethylated primers were 5′-TTTTGGGATTGTATTTGTTTTTGTTG-3′ and 5′-AAACAAAATACAAACCATATCCCCA-3′; ER methylated primers were 5′-TTTTGGGATTGTATTTGTTTTCGTC-3′ and 5′-AACAAAATACAAACCGTATCCCCG-3′. Both rounds of PCR were performed under the same conditions, with 2 μL of a 50-fold dilution of the first-round PCR product serving as the template for the second-round MSP. Amplification was performed under the conditions described above. PCR products were subjected to electrophoresis on 2% agarose gels.

### Chromatin immunoprecipitation assay

Chromatin immunoprecipitation (ChIP) assay was performed using the Magna ChIP Assay Kit(Millipore) according to the manufacturer's instructions. DNA cross-linking was performed by adding 1% formaldehyde into cell cultures at room temperature for 10 min, and glycine was then added (0.125 M final concentration) for 5 min to stop the cross-linking reaction. Cells were lysed with a lysis buffer with protease inhibitors and sonicated to shear genomic DNA to lengths between 200 and 800 bp. One-tenth of the cell lysate was used for input control and the rest was used for immunoprecipitation using antibodies against DNMT1. After collecting immunoprecipitates using protein G agarose columns, protein–DNA complexes were eluted and heated at 62 to reverse the cross-linking. After digestion with proteinase K, DNA fragments were purified by spin columns and analyzed by PCR. The ER promoter was analyzed using the 5′-primer 5′-AGTTGTGCCTGGAGTGATG-3′ and the 3′-primer 5′-GCAGAAGGCTCAGAAACC-3′. Initially, PCR was performed with different numbers of cycles or dilutions of input DNA to determine the linear range of the amplification; all results shown fall within this range. After 30 cycles of amplification, PCR products were run on 2% agarose gel and analyzed by ethidium bromide staining. All ChIP assays were performed at least twice with similar results.

### Immunohistochemistry

Immunohistochemical staining was performed on formalin-fixed, paraffin-embedded tumor samples. Sections mounted on silanized slides were dewaxed in xylene, dehydrated in ethanol, boiled in 0.01 M citrate buffer (pH 6.0) for 20 min in a microwave oven and then incubated with 3% hydrogen peroxide for 5 min. After washing with PBS, sections were incubated in 10% normal BSA for 5 min, followed by overnight incubation with rabbit anti-human ERα polyclonal antibody (1∶500), followed by anti-rabbit horseradish peroxidase–conjugated secondary antibody at room temperature for 30 minutes. The sections were then counterstained with hematoxylin, dehydrated, cleared, and mounted.

### Statistical analyses

The data were recorded as means ± SD. Statistical comparisons between control and treated groups were performed using either Student's t test or single-factor ANOVA model. p<0.05 was considered to indicate a statistically significant difference.

## Results

### Arsenic trioxide induced re-expression of ERα mRNA and protein in ER-negative human breast cancer cells

**Figure 1:**
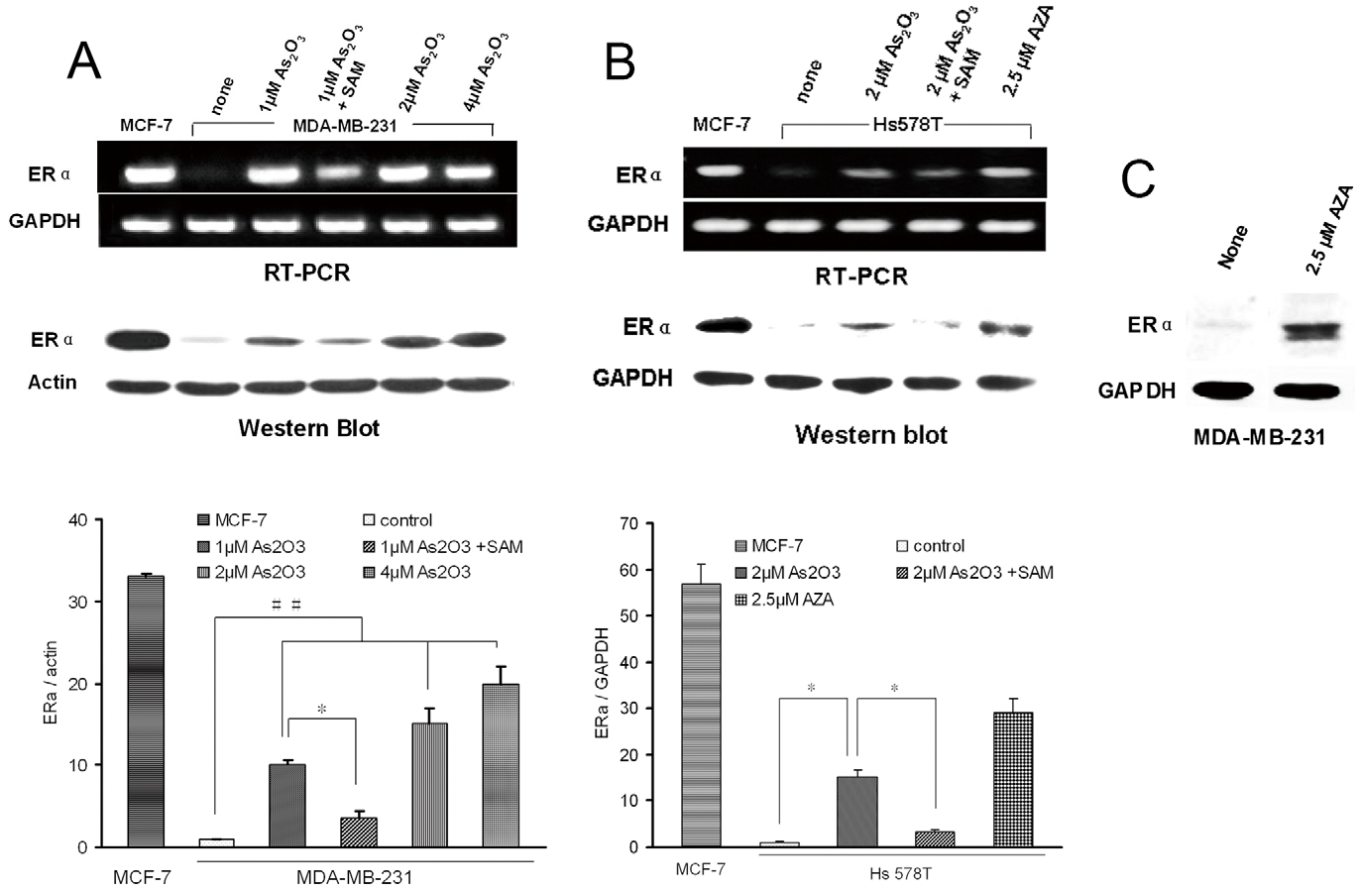
ERα mRNA and protein re-expression induced by arsenic trioxide in MDA-MB-231 cells (A) and Hs578T cells (B). ERα protein re-expression induced by AZA (2.5 μmol/L for 4 days) in MDA-MB-231 cells (C). RT-PCR and Western blotting analysis showed the re-expression of ER mRNA and protein after exposure of cells to arsenic trioxide (1, 2, 4 μmol/L for 6 days), whereas SAM (200 μmol/L for 6 days) reduced the degree of re-expression. The ER-positive prototype, MCF-7, was used as a positive control; untreated ER-negative MDA-MB-231 cells were used as a negative control. GAPDH and β-Actin provided a control for the amount of intact RNA and protein used in the reactions. ## *P*<0.01 compared with control cells. # *P*<0.05 compared with control cells. *P<0.05 compared with cells exposed to 1 μM arsenic trioxide.

MDA-MB-231 and Hs578T cells were used as a model of ER-negative breast cancers. These human cells are particularly suitable for preclinical studies because they are highly aggressive [13]. MCF-7 cells served as a positive control. RT-PCR and western blotting analysis revealed abundant ERα mRNA and protein in MCF-7 cells but none in MDA-MB-231 (Fig. 1A) and Hs578T cells (Fig. 1B). Exposure of MDA-MB-231 and Hs578T cells to arsenic trioxide for 6 days induced re-expression of ERα mRNA and protein. The methyl donor, SAM, reduced arsenic trioxide-induced up-regulation of ERα mRNA and protein expression. Furthermore, we also observed the re-expression of ERα protein in MDA-MB-231 cells (Fig. 1C) and Hs578T cells (Fig. 1B) treated with a known demethylating agent 5-aza-dC alone. These data are consistent with the concept that demethylation of the ERα promoter is linked to arsenic trioxide-mediated ERα re-expression.

### Arsenic trioxide induced restoration of ERα function in ER-negative human breast cancer cells

**Figure 2:**
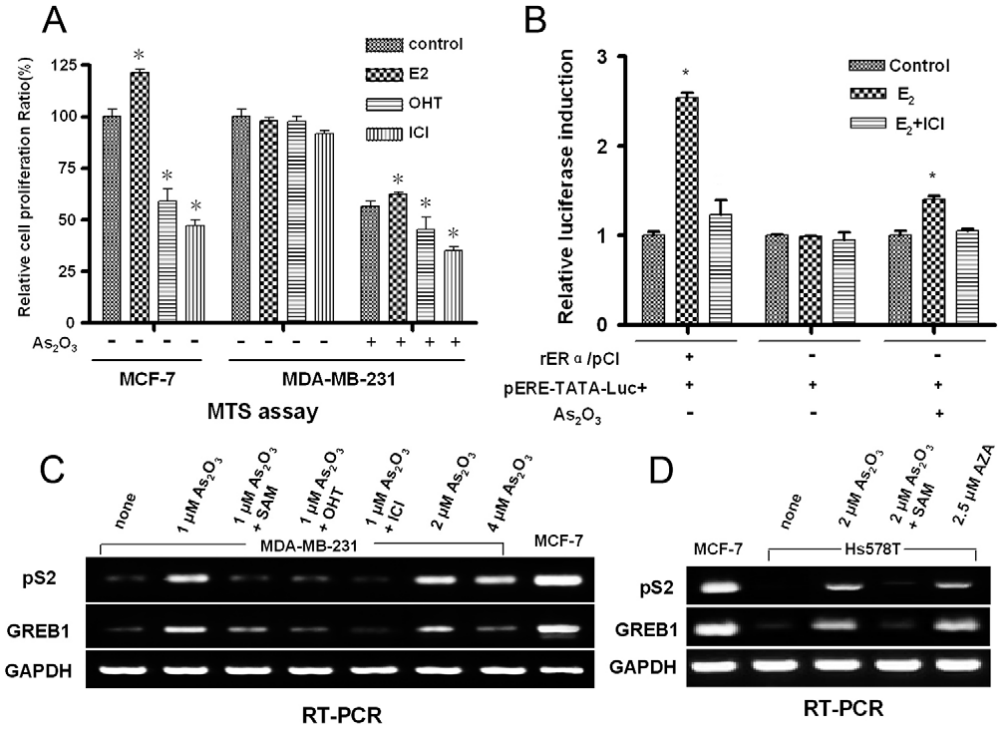
Re-expression of functional ERα in ER-negative MDA-MB-231 and Hs578T cells by arsenic trioxide. (A) Effect of arsenic trioxide (2 μmol/L for 6 days), E2 (10 nmol/L for 24 hr), OHT (10 μmol/L for 24 hr), and ICI (2 μmol/L for 24 hr) on cell growth was determined by MTS. MCF-7 and MDA-MB-231 cells were exposed to E2, OHT or ICI for 24 hr before assay. In addition, MDA-MB-231 cells were exposed to arsenic trioxide (2 μmol/L for 6 days), as described, along with E2, OHT or ICI for 24 hr before MTS assay. This experiment was repeated twice with similar results. (B) The transcriptional activities of re-expressed ERα was examined in MDA-MB-231 cells by luciferase reporter gene assay. Cells were transiently co-transfected with 0.5 μg of pERE-TATALuc+, 0.2 μg of rERa/pCI, and 0.1 μg of phRL-tk as an positive control; untreated MDA-MB-231 cells were transiently co-transfected with 0.5 μg of pERE-TATALuc+ and 0.1 μg of phRL-tk as a negative control. MDA-MB-231 cells were exposed to arsenic trioxide alone (2 μmol/L for 6 days), or in combination with E2 (10 nmol/L) or ICI (2 μmol/L) for 24 hr. The cell lysates were analyzed by use of the Dual-Luciferase Reporter Assay System kit. Ethanol solvent was used as control, and transcriptional activity was presented as fold of control. Values are presented as the means ± SD of three independent experiments. (C) Expression of ERα and its target genes (pS2 and GREB1) in MDA-MB-231 cells. Before RT-PCR was performed, cells untreated or pretreated with arsenic trioxide were exposed to SAM (200 μmol/L for 6 days), OHT (10 μmol/L for 24 hr), ICI (2 μmol/L for 24 hr), or vehicle in fresh estrogen-free medium. This experiment was repeated twice with similar results. **(**D) Expression of ERα target genes pS2 and GREB1) in Hs578T cells. Before RT-PCR was performed, cells untreated or pretreated with arsenic trioxide were exposed to SAM (200 μmol/L for 6 days), AZA (2.5 μmol/L for 4 days), or vehicle in fresh estrogen-free medium. This experiment was repeated twice with similar results.

Estrogen exerts its effects by binding to ER, which functions as transcription factor controlling cell proliferation and differentiation [14], [15]. As determined by MTS assays, proliferation of ER-positive MCF-7 cells was stimulated by exposure to E2 and inhibited by exposure to OHT or ICI. On the other hand, none of these treatments affected the proliferation of the ER-negative MDA-MB-231 cells (Fig. 2A). After exposure of the cells to 2 μmol/L of arsenic trioxide, the response of MDA-MB-231 cells to E2, OHT and ICI was restored, although the degree of growth stimulation/inhibition was less than that of MCF-7 cells.

To evaluate further the transcriptional activity of re-expressed ERα in ER-negative cells, the plasmid pERE-TATA-Luc+ and the rat ERα expression vector rERa/pCI were transiently co-transfected into ER-negative MDA-MB-231 cells as positive controls. The plasmid phRL-tk was used as internal control for transfection efficiency and cytotoxicity. As shown in Fig. 2B, E2 induced a 2.5-fold enhancement in luciferase activity in MDA-MB-231 cells transfected with pERE-TATA-Luc+ and rERa/pCI and exposed to 100 nM E_2_ for 24 hr (significantly different from control, P<0.05). The ER antagonist ICI reduced E_2_-induced luciferase expression. ER-negative cells transfected only with pERE-TATA-Luc+ and exposed to E_2_ or ICI showed no significant alterations. ER-negative cells exposed to 2 μmol/L of arsenic trioxide were likewise responsive to the stimulatory effect of E2; the effect of arsenic trioxide on ERα-regulated transactivation was also inhibited by ICI. These results are consistent with the observation that arsenic trioxide pretreatment of ER-negative cells restored ERα mRNA and functional ERα protein.

To establish that the re-expressed ERα in MDA-MB-231 and Hs578T cells is a functionally active transcription factor that controls the expression of ER-responsive genes, the capacity of ligand-bound ER, through interaction with estrogen-response elements (EREs) in the DNA sequence, to activate expression of the estrogen responsive genes, pS2 and GREB1 was determined. In E2 exposed ER-positive MCF-7 cells, the ER-responsive genes were expressed, whereas MDA-MB-231 (Fig. 2C) and Hs578T (Fig. 2D) cells only showed low levels of expression of these genes. Re-expression of ERα induced by arsenic trioxide in ER-negative cells was associated with increased expression levels of the ER-responsive genes, pS2 and GREB1, in the presence of E_2_, and their expression was inhibited by SAM, OHT or ICI (Fig. 2C and 2D). As expected, the expression of pS2 and GREB1 were upregulated following exposure of demethylating agent 5-aza-dC in ER-negative cells (Fig. 2D).

### Arsenic trioxide induced functional re-expression of ERα in *in vivo*

**Figure 3:**
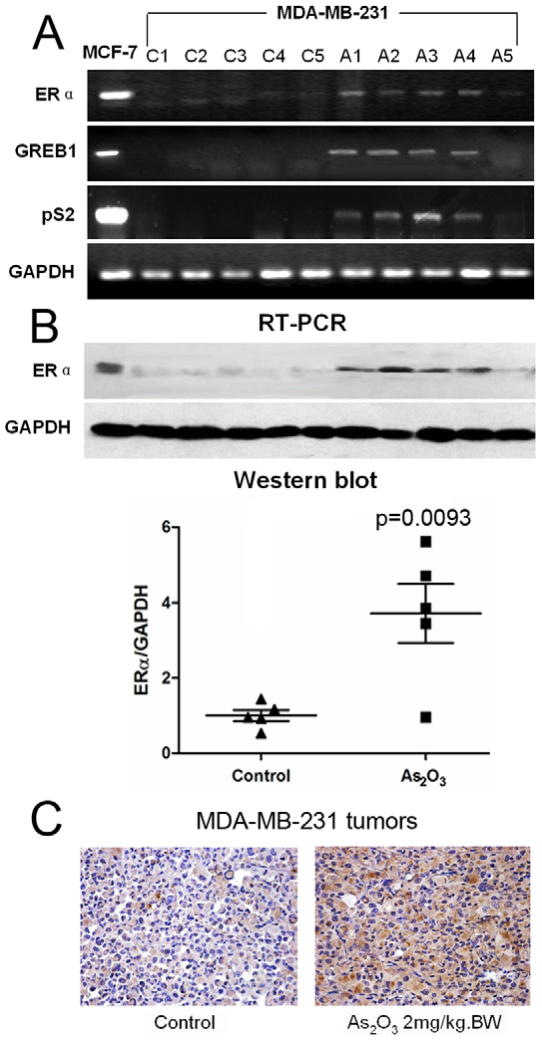
Arsenic trioxide induces functional re-expression of ERα *in vivo*. (A) Expression of ERα mRNA and its target genes (pS2 and GREB1) in MDA-MB-231 tumors. Arsenic trioxide (2 mg/kg BW) was administered i.p. in 100 μl of sterile saline for 4 weeks. The ER-positive prototype, MCF-7, was used as a positive control. C1–C5: control group, A1–A5: arsenic trioxide-treated group. (B) Expression of ERα protein in MDA-MB-231 tumors. The relative expression level of ERα protein is represented in a scatter plot. (C) Representative immunohistochemistry for ERα in MDA-MB-231 tumors using an anti- ERα polyclonal antibody.

Following the illustration of arsenic trioxide-induced functional re-expression of ERα in *in vitro,* we further examined whether arsenic trioxide induces functional re-expression of ERα in an animal model. MDA-MB-231 cells were mixed with Matrigel and injected into the flank of nude mice. Arsenic trioxide (2 mg/kg.bw) was administered i.p. in 100 μl of sterile saline for 4 weeks. We observed that treatment of MDA-MB-231 tumors to arsenic trioxide resulted in significant re-expression of ERα mRNA and protein (p = 0.0093) in four out of five mice, as measured by RT-PCR and Western blot analyses (Fig. 3A and 3B). Re-expression of ERα protein by arsenic trioxide treatment was further demonstrated by immunohistochemical staining (Fig. 3C). Expression of the ER-responsive genes, pS2 and GREB1 were also induced by arsenic trioxide in ER-negative tumors (4/5) (Fig. 3A). These *in vivo* results further confirm our *in vitro* findings. Taken together, these data show that arsenic trioxide significantly induces functional re-expression of ERα in an *in vivo* model.

### Arsenic trioxide-induced demethylation of ER promoter CpG island

**Figure 4:**
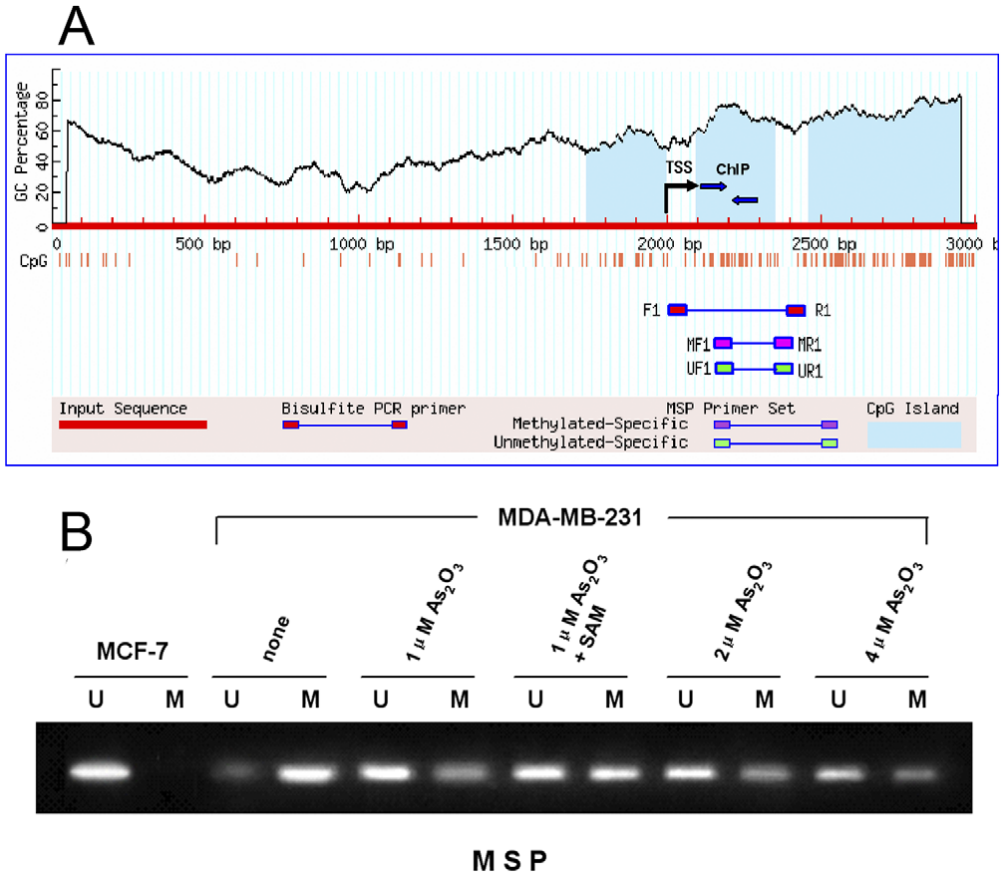
Methylation analysis of the ERα promoter by MSP in MDA-MB-231 cells. (A) A 2.5 kb genomic sequence of the ERα promoter, analyzed by the online program, www.urogene.org/methprimer, revealed the presence of a high GC percentage CpG islands (blue) between relative positions 2000 and 2500. Position 2000 indicates the transcription start site (TSS, arrow). A region of high CpG (red vertical bars) density was chosen for MSP analysis. This region includes 28 CpG dinucleotides. (B), Methylation analysis of the ERα promoter by MSP in breast cancer cell lines. Sensitivity of the utilized MSP primers was determined by a dilution series of methylated DNA with unmethylated DNA. At least 1% of methylated DNA (∼0.1 ng) can be detected with the ER MSP primers. In each set, M primer pairs anneal only to sequences that are methylated before bisulfite treatment, whereas the U primer pairs anneal only to sequences that are unmethylated.

Since demethylation of ERα promoter could induce the expression of ERα, it was still not clear whether that the re-expression of ERα induced by arsenic trioxide is also mediated through demethylation of the ERα promoter. This possibility was examined, for bisulfite-treated DNA from breast cancer cells, by nested methylation-specific PCR (n-MSP), the most sensitive method for determining methylated states. A region of 2000 bp of the ERα promoter and 500 bp downstream of the annotated transcription start site (TSS) was analyzed by Methyl Primer Express software to design MSP primers. This region was also analyzed by the online program, MethPrimer (www.urogene.org/methprimer), to design bisulfite sequencing PCR (BSP) primers. The primers for BSP and MSP covered the same region, 500 bp downstream of the annotated TSS, a region of high CpG density and partial overlap (Fig. 4A).

n-MSP was performed by two rounds of PCR as described in Materials and Methods. DNA bands in lanes labeled with U indicate MSP products amplified with primers recognizing the unmethylated promoter sequence (Fig. 4B). DNA bands in lanes labeled with M represent amplified MSP products with methylation-specific primers. DNA from untreated MDA-MB-231 cells yielded a PCR product only with methylation-specific primers, whereas DNA from MCF-7 cells yielded a PCR product only with unmethylation-specific primers. Exposure of cells to increasing concentrations of arsenic trioxide led to decreasing levels of the PCR product corresponding to methylated CpG islands. SAM (200 μmol/L) restored the methylated state of CpG islands in the ERα promoter.

**Figure 5:**
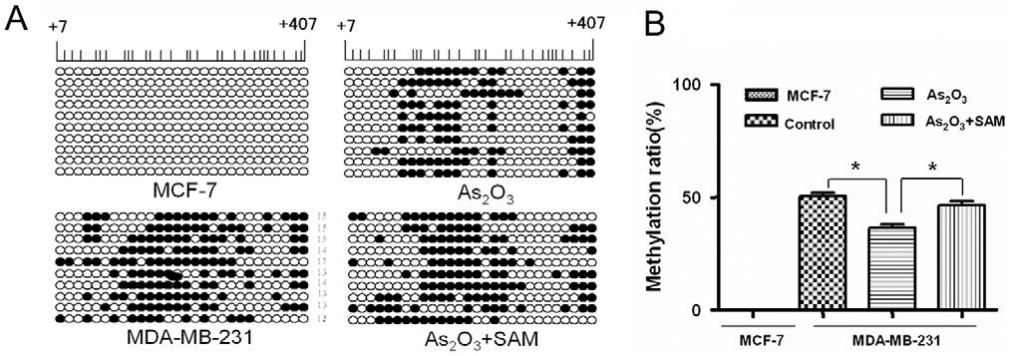
Methylation analysis of the ERα promoter by BSP in breast cancer cells. (A)Shown is a representative bisulfite sequence analysis for the high CpG region of the ERα promoter, which contains 28 CpG dinucleotides. For MDA-MB-231 and MCF-7 cell lines (10 replicates each), methylated CpGs are designated by closed circles; unmethylated CpGs are designated by open circles. (B)Statistical chart of methylation analysis; the results shown represent the means ± S.E. of three independent experiments. Significant differences from the controls as determined by Student's t test are indicated by asterisks (*P* <0.05).

To corroborate this conclusion, bisulfite genome sequencing PCR (BSP) was performed to measure methylation status. The status of individual CpG sites was determined by comparison with the sequence from known ERα gene sequences. With bisulfite modification, originally unmethylated CG changes to TG, whereas methylated CG does not change. As shown in Fig. 5A, in MCF-7 cells there are mostly TGs, but CGs are predominant in MDA-MB-231 cells. This is consistent with the MSP results and indicates that the ERα promoter is mostly unmethylated in MCF-7 cells but mostly methylated in MDA-MB-231 cells. After cellular exposure to 2 μmol/L of arsenic trioxide, most CGs changed to TGs. This indicates that, in MDA-MB-231 cells, the ERα promoter is demethylated. In the presence of SAM, some of the TGs changed into CGs.

The region sequenced contains 28 CpG dinucleotides, indicated by circles. CpG islands of MCF-7 were completely unmethylated. In contrast, the ER-negative MDA-MB-231 cells were hypermethylated. In MDA-MB-231 cells, there was partial demethylation of CpG islands after exposure to 2 μmol/L arsenic trioxide. The combination of arsenic trioxide and SAM partially restored the methylation status of MDA-MB-231 cells. The values and statistical differences are expressed in following figure (Fig. 5B). The results confirmed that arsenic trioxide-induced demethylation of the ERα promoter is random, not site-specific.

### Arsenic trioxide altered expression of DNMTs and MBD2 proteins in MDA-MB-231 cells

**Figure 6:**
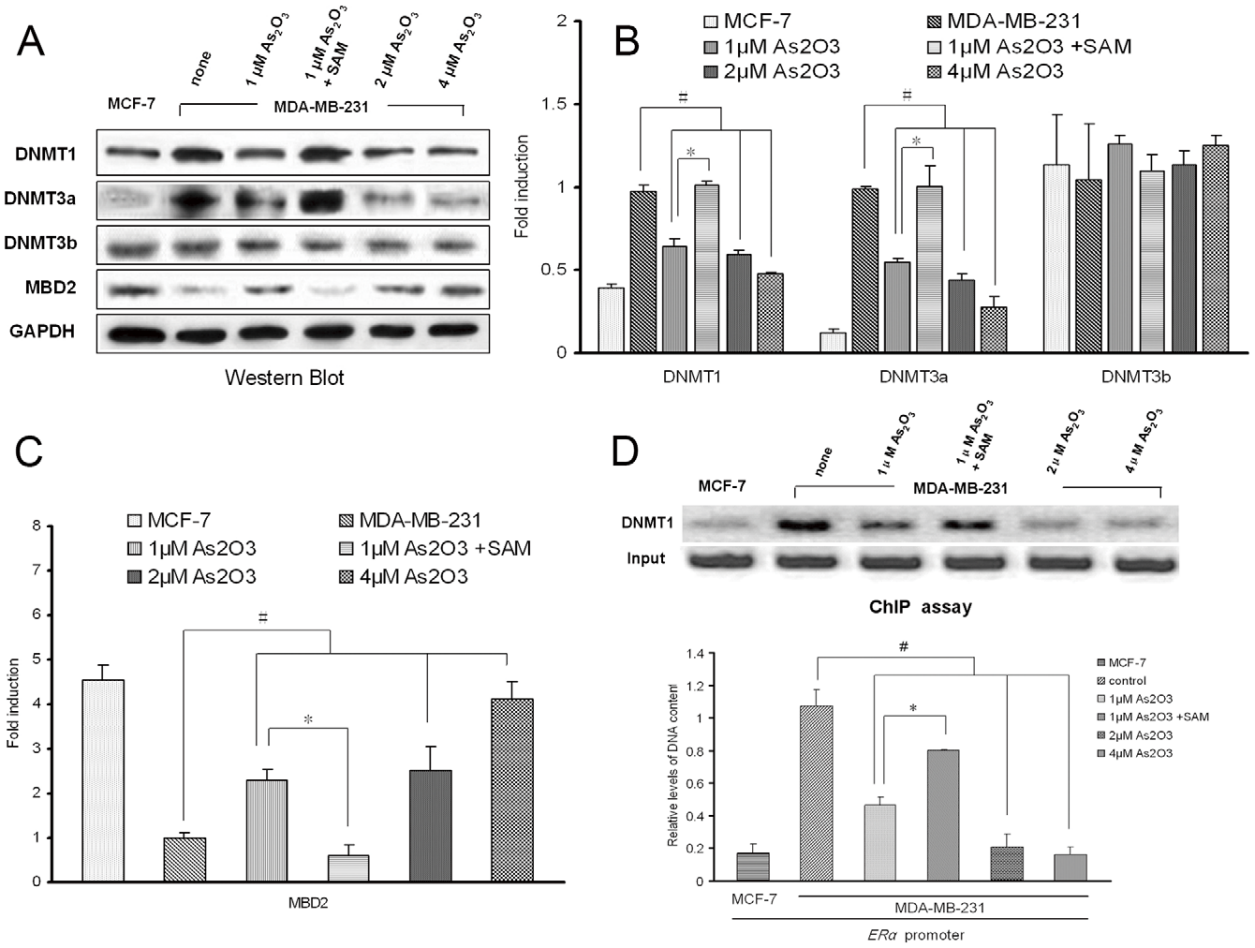
Methylation-related proteins mediate arsenic trioxide-induced re-expression of ERα. (A) Immunoblot analysis of DNMTs and MBD2 protein. Equal amounts of protein (80 μg) from whole-cell lysates of the control MCF-7 and MDA-MB-231 cells, treated as in Fig. 1, were separated by SDS-PAGE and subjected to Western blot analysis with specific antibodies against DNMT1, DNMT3a, DNMT3b and MBD2. Equivalence of protein loading was demonstrated by immunoblotting with anti-GAPDH antibody. (B) Relative protein levels (mean± SD) of DNMTs (n = 3). (C) Relative protein levels (mean± SD) of MBD2 (n = 3). (D) ChIP analysis of association of DNMT1 and ERα promoter. Cross-linked chromatin prepared from ER-positive MCF-7 and ER-negative MDA-MB-231 human breast cancer cells was immunoprecipitated with antibodies DNMT1. MDA-MB-231 cells treated as in Fig. 1. The immunoprecipitates were subjected to PCR analysis. Aliquots of chromatin taken before immunoprecipitation were used as input controls (n = 3). # *P*<0.05 compared with control cells. *P<0.05 compared with cells exposed to 1μM arsenic trioxide.

In ER-negative MDA-MB-231 cells, the methylated ER promoter is associated with DNMTs and methyl-binding proteins. We investigated whether treatment with arsenic trioxide alters the level of DNMTs and MBD2 protein expression. As showed in Fig. 6A–C, the expression levels of DNMT1 and DNMT3a protein in MDA-MB-231 cells was higher than in MCF-7 cells, meanwhile we found that exposure of MDA-MB-231 cells to arsenic trioxide for 6 days reduced DNMT1 and DNMT3a and increased MBD2 protein levels; cotreatment with SAM resulted in an opposite effect, and with increased concentration of exposure to arsenic, there was more down-expression of DNMT1 and DNMT3a and up-expresion of MBD2 protein. The level of DNMT3b protein was unaltered after arsenic trioxide and/or SAM treatments.

### Arsenic trioxide altered the association of DNMT1 with ERα promoter in MDA-MB-231 cells

We next determined the effects of arsenic trioxide on the association of DNMT1 with the ERα promoter using ChIP assays with specific DNMT1 antibody. ChIP assays revealed that DNMT1 protein is associated with the silenced ERα promoter in MDA-MB-231 breast cancer cells, whereas the active ERα promoter in MCF-7 cells shows little association of DNMT1 protein (Figure. 6D). Treatment of MDA-MB-231 cells with arsenic trioxide alone leads to a significant reduction in DNMT1 occupancy of the ERα promoter, and this was antagonized by co-treatment with SAM. Thus, reduction of DNMT1 protein expression along with partial dissociation of DNMT1 from the ERα promoter may be responsible for the ERα reactivation in cells exposed to arsenic trioxide.

## Discussion

Estrogen and its receptor are involved in the development and function of the mammary gland. Since the effects of endocrine therapy are primarily mediated through ERα, its expression is a predictor of response to treatment with selective estrogen receptor modulators (SERMs). Lack of ERα expression is the dominant mechanism for *de novo* resistance to 4-hydroxytamoxifen [16], [17], [18], [19]. In addition, some breast cancers that are initially ER-positive lose ERα expression during tumor progression and attain hormone unresponsiveness [3]. ER-negative tumors are more aggressive and are associated with poor histological differentiation and a higher growth fraction. New strategies for reactivation ERα and sensitization of ER-negative tumors to endocrine treatment are required. The mechanism for silencing ERα in the ER-negative MDA-MB-231 cells is not clear. Although mechanisms for the lack of ERα expression in breast cancer have been investigated [20]; hypermethylation of the ERα promoter represents the first identified mechanism. The present study provides evidence that exposure of ER-negative breast cancer cells to arsenic trioxide leads to re-expression of ERα mRNA and the ERα protein. Nevertheless, the level of expression is lower than in MCF-7 cells, which are ER-positive, indicating that there could be other mechanisms involved in silencing of ERα. Other epigenetic events, such as deacetylation and methylation of histones, are involved in the regulation of promoter transcription. Exposure to inhibitors of HDACs, including trichostatin A and vorinostat, lead to re-expression of ERα in ER-negative cells such as MDA-MB-231 [21].

Arsenic trioxide is effective in treating acute promyelocytic leukemia, and its therapeutic effects on solid tumors are being evaluated [9]. Arsenic trioxide could suppress cancers of the liver, prostate, and esophagus through demethylation or, for example, by induction of apoptosis. Zhang et al recently showed that arsenic trioxide induces re-expression of ER in MDA-MB-435s and also inhibits tumour growth in mice [22]. However, there is evidence that MDA-MB-435 cell line is not actually from a breast cancer lineage, but in fact are derived from a melanoma cell line, M14 [23]. Zhang's paper used MDA-MB-435s cell line, which is a derivative of the parent MDA-MB-435 cell line, and therefore is also a melanoma lineage. Thus, the possible use of arsenic trioxide for the treatment of ER-negative breast cancer has not previously been reported.

Arsenic trioxide, as an endocrine-disrupting chemical, act as hormone mimetic and as either agonist or competitive antagonist dependent on the level of endogenous estrogens. It is now generally accepted that ER protein is targeted for rapid degradation via the ubiquitin- proteasome pathway in response to E2 in breast cancer cells. Stoica et al demonstrate that arsenic trioxide mimics the effects of E2 in the ERα-positive human breast cancer cell line MCF-7 [24]. Chow et al also found that arsenic trioxide could down-regulate ERα mRNA and protein levels [25]. Different from Stoica's study, Chow et al found that arsenic trioxide abolished transcriptional induction of the estrogen responsive gene pS2 mediated by E2. Arsenic trioxide exhibits anti-estrogenicity on MCF-7 cells. The different results of Stoica and Chow are likely a result of one or more differences in experimental culture medium. Arsenic trioxide can act as an agonist for ER [24], activating it in the absence of hormone(phenol red-free IMEM supplemented with 5% charcoal stripped calf serum). However, arsenic trioxide can act as an antagonist for ER [25], inhibiting it in the presence of hormone (RPMI 1640 medium supplemented with 10% dextran-coated charcoal- stripped FBS). It is well established that phenol red in culture medium is estrogenic. In our study, the medium we used was similar to that of Stoica's study (phenol red–free DMEM supplemented with 10% CS-FBS), which was estrogen-free. In this medium, arsenic trioxide induced the re-expression of ERα through demethylating, it also can act as an agonist for ER, activating ER via a mechanism similar to Stoica's study(ERα protein degradation). Combining the effects of demethylation and estrogenicity, arsenic trioxide induces a weak expression of ER protein in ER-negative breast cancer cells.

ERα, a predictor of prognosis and response to endocrine therapy, functions in development of normal breast tissue. It has also been linked to mammary carcinogenesis, breast tumor progression, and outcomes for breast cancer patients [26]. To date, the molecular mechanisms underlying the loss of ERα in breast cancer have been poorly understood. Hypermethylation of the CpG island in the ERα promoter is known to be responsible for silencing of ERα expression in ER-negative breast tumors [19]. In the present report, the state of CpG islands in ER-positive breast cancer cells and ER-negative breast cancer cells was examined by two methods, MSP and BSP. Our results demonstrate that the CpG islands of MCF-7 were completely unmethylated in contrast with the ER-negative MDA-MB-231 cells, which were hypermethylated, and our results demonstrate that exposure of MDA-MB-231 cells to arsenic trioxide alone leads to a significant reduction in the extent of CpG dinucleotide methylation in comparison with untreated MDA-MB-231 cells. This suggests that arsenic trioxide induces demethylation of the ERα promoter. This compound induces apoptosis and has growth-suppressive effects as a treatment for acute promyelocytic leukemia and solid tumors [27], [28], [29], [30]. But its effects on demethylation are still few [31], [32], [33]. Our results confirm that arsenic could act as a demethylation agent, and demethylation of ERα promoter induced by arsenic trioxide is not site-specific.

Arsenic trioxide reactivates functional ERα and also induces demethylation of the ERα promoter. Thus, we hypothesize that arsenic trioxide reactivates ERα through demethylation of the ERα promoter, which is silent due to hypermethylation. Since both the metabolism of arsenic and DNA methylation require SAM as a methyl donor [34], [35], the consumption of methyl groups in arsenic biotransformation presumably results in a deficiency of methyl donors, reducing DNA methylation [10]. This hypothesis was evaluated by treatment of cells with the combination of arsenic trioxide and SAM. If arsenic trioxide-induced DNA demethylation is a consequence of lack of a methyl donor, the effect should be reversed by SAM, as observed in this study. The results of BSP and MSP analysis support a direct association between ERα silencing and the hypermethylation status of the ER promoter. Additionally, SAM reverses ERα demethylation and the ERα re-expression induced by arsenic trioxide. These results confirm our hypothesis that the reactivation of silent ERα in ER-negative breast cancer cells is due to demethylation of the ERα promoter by arsenic trioxide.

Arsenic methylation requires SAM as a cofactor and, as yet, largely uncharacterized methyltransferases (MeTases), including DNA MeTases (DNMTs), a group of enzymes responsible for DNA methylation. Expression of the DNMT gene has been definitively linked to methylation status [36], [37]. Arsenic trioxide, an inhibitor of sulfhydryl enzymes, inhibits the activity of DNMT3a and DNMT3b, both of which contain cysteine sulfhydryl groups [38], [39]. It may function by inhibiting de novo methylation. Alternatively, with continuous exposure to a methylation substrate such as arsenic, cells could produce greater quantities of S-adenosylhomocysteine (SAH), the byproduct of methylation with SAM as the methyl donor. SAH is a competitive inhibitor of DNA methyltransferases activity [40], [41]. In the current study, expression of the DNMTs protein was measured. The results are similar to those of reports that arsenic trioxide inhibits DNMT1 protein in human liver and colon cancer cells [42], [43], we found that arsenic trioxide also inhibits DNMT3a protein in ER-negative breast cancer cells. These results confirm that arsenic trioxide metabolism consumes the methyl donor, SAM, and inhibits DNMTs. Thus, factors involving arsenic metabolism contribute to the level of DNA methylation in arsenic-induced demethylation of the ERα promoter. In addition, ChIP assays validated that DNMT1 was associated with ERα promoter in MDA-MB-231 but not MCF-7 cells, treatment of MDA-MB-231 cells with arsenic trioxide alone leads to a significant reduction in DNMT1 occupancy of the ERα promoter, the partial dissociation of DNMT1 from ERα promoter was benefit for reactivation of silented ERα promoter. It was also antagonistic by cotreatment with SAM. Thus, arsenic trioxide inhibition of total DNMT1 protein along with partial dissociation of DNMT1 from the ERα promoter facilitates ERα promoter demethylation and reactivation.

In summary, our results identify a mechanism for ERα reactivation by arsenic trioxide, which, through competition with SAM for methylation of DNA and inhibition of DNMT1 protein along with partial dissociation of DNMT1 from the ERα promoter, causes demethylation of the ERα promoter in ER-negative breast cancer cells. Furthermore, restoration of ERα expression by arsenic trioxide is sufficient to induce anti-estrogen responses in ER-negative breast cancer cells and animal model. A better understanding of the regulatory mechanisms governing ER gene silencing will contribute to the design of combined therapies and to innovative strategies for drug delivery.
